# Letter from the Editor in Chief

**DOI:** 10.19102/icrm.2023.14032

**Published:** 2023-03-15

**Authors:** Moussa Mansour


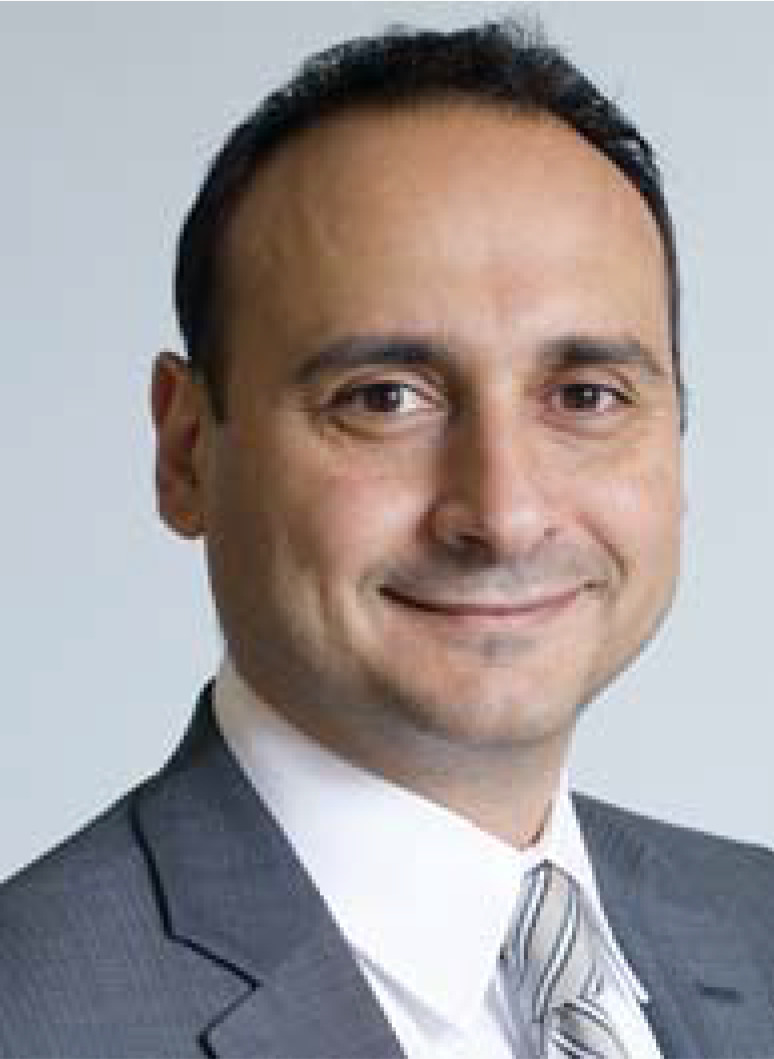


Dear readers,

During the annual scientific meeting of the American College of Cardiology earlier this month, the Pulsed Field Ablation for the Treatment of Atrial Fibrillation: PULSED AF Pivotal Trial was presented by Verma et al. as a late-breaking clinical trial with simultaneous publication in *Circulation*.^1^ PULSED AF is a single-arm prospective multinational clinical study designed to evaluate the effectiveness and safety of the novel PulseSelect pulsed field ablation system (Medtronic, Minneapolis, MN, USA) in patients with paroxysmal or persistent symptomatic atrial fibrillation (AF) during a 12-month follow-up period at 41 centers in 9 countries. The PulseSelect catheter is an over-the-wire circular catheter with 10 electrodes allowing bipolar biphasic ablation. All enrolled patients had failed or were intolerant to class I or III anti-arrhythmic medications. The primary effectiveness endpoint was freedom from a composite endpoint of acute procedural failure, arrhythmia recurrence, or anti-arrhythmic escalation through 12 months. The primary safety endpoint was freedom from a composite of serious procedure- and device-related adverse events. The study also had pre-specified secondary endpoints, including a clinical effectiveness endpoint defined as freedom from symptomatic atrial arrhythmias. Both primary effectiveness and safety endpoints were met. Pulsed field ablation was shown to be effective at 1 year in 66.2% (95% confidence interval [CI], 57.9–73.2) of patients with paroxysmal AF and 55.1% (95% CI, 46.7–62.7) of patients with persistent AF. The primary safety endpoint occurred in just 1 patient (0.7%; 95% CI, 0.1–4.6) total among both the paroxysmal and persistent AF cohorts.

The PULSED AF study has some limitations, including the fact that it was non-randomized. Another limitation is its heterogeneous population that includes both paroxysmal and persistent AF cases, which is probably the reason for the continuation of anti-arrhythmic medications during follow-up. Nevertheless, this is an important study for many reasons. First, the safety result in PULSED AF was excellent, and, while not novel, it confirmed the findings of prior studies demonstrating that pulsed field ablation does not, or is significantly less likely to, cause the most feared complications of thermal AF ablation like esophageal injury and pulmonary veins stenosis. Second, PULSED AF is the first investigational device exemption study for pulsed field ablation conducted in the United States, and it marks the beginning of a new and exciting era in the field of AF ablation. As for effectiveness, the success rate in PULSED AF falls into the same range as rates from studies using thermal ablation. It is expected that, with operator experience and new-generation devices, the success rate will improve in future studies.

I hope that you enjoy reading this issue of *The Journal of Innovations in Cardiac Rhythm Management*.


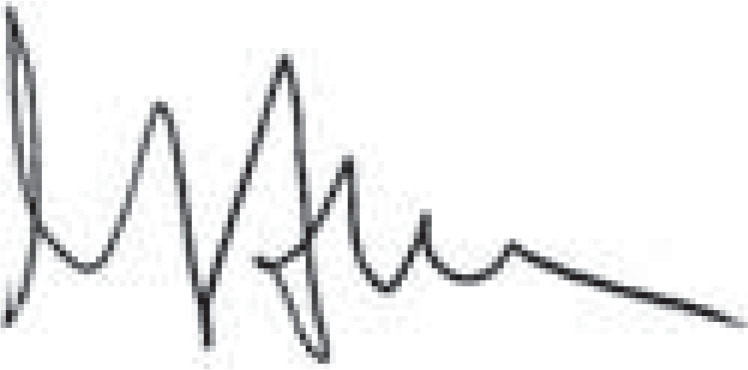


Sincerely,

Moussa Mansour, md, fhrs, facc

Editor in Chief

*The Journal of Innovations in Cardiac Rhythm Management*

MMansour@InnovationsInCRM.com

Director, Atrial Fibrillation Program

Jeremy Ruskin and Dan Starks Endowed Chair in Cardiology

Massachusetts General Hospital

Boston, MA 02114
